# Atlanta Academy of Medicine

**Published:** 1877-02

**Authors:** J. S. Todd

**Affiliations:** Reporter


### Reports of Societies.

### ATLANTA ACADEMY OF MEDICINE.

J. S. TODD, M.D., Reporter.

Atlanta, Dec. 19, 1876.

Academy called to order by Dr. Miller, President.

The reports of cases being in order, Dr. J. G. Westmoreland desired to report an obstetrical case, which, though a natural labor, presented a larger number of surmountable causes of delay than is usually met with in one case. He thinks that while officious interference is always to be deprecated, yet that there are many slight difficulties retarding the progress of labor, some of which may be readily removed, and thus save the patient much time of suffering.

The case referred to was a second labor, and, counting from the last menstrual period, the time of gestation had been extended to ten months. The size of the child and the unyielding condition of the cranial bones, confirmed this opinion. This, with a want of force in the uterine contractions, particularly of the longitudinal fibers, delayed progress materially. The first could not, of course, be remedied; but finally, when more expulsive contractions seemed to be demanded, ergot was given, and followed promptly with more vigorous throes. An anterior inclination of the uterus throwing the os nearer the sacrum not only directed the expulsive force too much posteriorly toward the coxcyx and perineum, but allowed the anterior lip of the womb to remain much below the pubis and in front of the anterior portion of the head, even when the latter was pressing against the perineum posteriorly. These causes of delay were to some extent obviated by constant gentle pressure anteriorly against the fundus with one hand, while with a finger of the other the anterior œdemitous lip of the os was pressed firmly up under the pubis, in the absence of, as well as during a pain. When the sack of water filled the vagina and pressed forcibly against the perineum, no perceptible progress was made for several pains, manifestly on account of unusual strength of the membranes. In proof of this, after rupturing them, the head was found to advance rapidly.

Dr. Miller thought that cases of protracted gestation were occasionally met with. The difficulty of fixing absolutely the time of conception rendered uncertain the duration of pregnancy, and often when the woman goes beyond the term of nine months according to the count from the last menstrual period, it is attributed to an error in concluding that conception occurred immediately succeeding the last menstruation instead of three weeks later, as is supposed sometimes to be the case. He said the opinion of protracted term is so well established that in some nations, among them France, legal enactments recognize the possible extension to ten months or three hundred days. He believes there is a tendency to abort or to delivery after maturity at periods of exact multiples of twenty-eight days from the commencement of the last regular menstruation, more than at intermediate periods. So that abortion is likely to occur at the expiration of twenty-eight, fifty-six, a hundred and twelve, etc., and the delivery of a mature child at two hundred and eighty or three hundred and eight days, more than at any intermediate time. In regard to the condition of the anterior lip of the womb, mentioned by Dr. Westmoreland as somewhat interfering with the progress of labor, he remembers to have met occasionally with some hindrance to advancement of the head, and generally pursued the course just mentioned for relief. Has also met frequently with toughness of the membranes as just described, in which rupturing accelerated labor.

Dr. Armstrong thought he had witnessed cases of protracted gestation.

Dr. W. F. Westmoreland reported a death from septicæmia following the removal of a large ovarian tumor. Also the peculiarity of a case of stone, in which after the lateral operation of lithotomy no urine escaped through the cut. He at first felt uneasy lest infiltration was occurring from its passing out of the bladder and not through the external opening, but ascertained that the cut in the bladder was closed by unusual contraction of the viscus. Notwithstanding the cut in the bladder be small as in this case, he expects dribbling always except after the median operation. Usually allows the urine to pass through the cut in preference to keeping a catheter in the bladder, which he thinks a source of irritation.

Dr. Miller, while he does not claim equal experience and judgment on this subject, always keeps a catheter in the urethra for the discharge of the urine under such circumstances, and without evidences of irritation from it.

Dr. Armstrong reported two cases of diphtheria with good recovery, in which diphtheretic exudation or false membrane was distinct.

Dr. J. G. Westmoreland alluded to the practical importance of settling the question as to the contagiousness of this disease. He had met with no satisfactory evidence, in his practice, of it having been communicated from one person to another. Thinks that, like influenza, it may prevail as an epidemic, attacking whole families and pervading an entire neighborhood.

Dr. W. F. Westmoreland relied on the experience of those who had seen it prevail extensively, and who obtained satisfactory evidence of its contagious character, and had even known of its communication to lower animals. Thinks that all forms of sore throat, where even an apparent pseudo-membrane is found, are not diphtheria, as supposed, and hence the unbelief in its contagious character.

Dr. Baird had no satisfactory reason for believing the disease contagious. Had known diphtheretic exudation brought in contact with the fauces of persons without communicating the disease. He does not have any confidence in the German theory of bacterial origin, and thinks bacteria probably the result of pustulation in the fibrinous exudation.

Dr. Todd's experience with diphtheria did not impress him with the idea of contagion.

Dr. Armstrong was not settled in opinion on this subject, but alluded to the importance of being able to decide this question.

Academy adjourned.

---

Atlanta, Jan. 9, 1877.

The President, Dr. V. H. Taliaferro, in the chair.

Dr. Baird read an essay on "Cerebral Embolism," which he defined to be the sudden closure of a cerebral artery by an embolus or clot formed in some portion of the body, and carried by the current of the circulation into a smaller vessel, where it became impacted, completely arresting the circulation beyond the point of obstruction. He referred to the origin and distribution of the cerebral arteries, and contended that the angle at which the left carotid was given off from the aorta favored the entrance of migrating plugs into that vessel, and as a result the hemiplegia of embolism was, in the great majority of cases, on the right side of the body. The immediate local effect of cerebral embolism is anæmia of the territory to which the occluded vessel is distributed, and the morbid process involved, except where the collateral blood supply is speedily established, is briefly told—anæmia, necrosis, softening and disintegration. From the nature of the cause, the access of the affection is always sudden. The paralysis, when it occurs, may involve a whole side, or be limited to a single muscle or group of muscles; sometimes there is no paralysis, but simply loss or impairment of the faculty of language.

Endo-carditis, the result of acute articular rheumatism, occupies the most important causative relation toward cerebral embolism. The collections that form on the valves of the heart being liable to wash off, and result in this affection. Disintegration of the clots in aortic and carotid aneurisms and disintegrating thrombi may act in same way.

He referred in this connection to a case recently reported to the Academy by Dr. Logan, in which an aortic aneurism was the source of the embolus. Some fully devoloped cases may be mistaken for cerebral hemorrhage; the main points to be considered in making the discrimination are the invariable absence, in embolism, of premonitory symptoms, the pre-existence of valvular disease of the heart, *right* hemiplegia, and the age of the subject. The prognosis is always grave, and if rerecovery ensue, the liability to subsequent attacks should be borne in mind.

The treatment should be regulated by circumstances. Increase the amount of blood in the brain if the symptoms indicate deficiency, and diminish it if it becomes, as it frequently does, excessive. The patient should be kept quiet and comfortable, and when the head symptoms have disappeared, and he begins to make efforts to move his paralyzed limbs, faradic electricity should be employed.

Dr. Logan said there was a very decided improvement in the case of embolic hemiplegia reported by him at a previous meeting. He could now articulate with tolerable distinctness, and uses the right or paralyzed leg. He feels very much encouraged by the symptoms.

Dr. Miller: As a ligature thrown around the carotid (which was by no means an uncommon surgical operation) had never under his observation or reading caused hemiplegia, he was not prepared to admit that an occlusion of the artery from any other cause or causes would produce that result. Therefore he thought in Dr. Logan's case that the stoppage of the circulation in the carotid had nothing to do with the paralysis.

Dr. Baird said he had read of one case where tying of the common carotid had caused paralysis.

Dr. Miller asked how recently after the operation did paralysis take place.

Dr. Baird: It was only a tabulated case, recorded by Erichson in his Surgery. He agreed with Dr. Miller as to the vast majority of cases, and in this particular case referred the seat of the difficulty to the middle cerebral artery of the left side.

Dr. Miller said that the angle at which a smaller artery was given off from a larger, in his opinion, had nothing to do with the direction that would be pursued by a foreign body; it certainly did not influence the quantity of blood supplied to the various organs. The state of the function of the organ supplied by the vessels controls the amount of blood which they receive. Embolism of the cerebral arteries, while it does occur, is a much rarer accident from valvular disease of the heart than dropsy, for instance. And again, effusive or hemorrhagic paralysis are more frequent than embolic. According to Brown-Sequard, all people are ambidextrous when infants, but by habit they learn to use the right side in preference to the left. Hence the left side of the brain is more used than the right; more thinking is done by it; it is larger; its ganglia is more important; its blood supply greater; and he was not, therefore, puzzled to account for the greater frequency of disease of the left side of the brain.

Dr. Logan acquiesced in the views of Dr. Miller, and said that extreme tenderness and enlargement of the carotid led him to infer, doubtless erroneously, that its occlusion caused the paralysis. He still fails to detect pulsation in the left carotid.

Dr. Baird is certain that there was pulsation, or at least a flow of blood through the carotid when he examined the case. The impulse was much obstructed by the aneurism of the aorta below, and therefore difficult of detection. Of two hundred and forty post mortem examinations for disease of the brain of an embolic character, in only seventeen were emboli found on the left side. Said, while he agreed with Dr. Miller that it was as easy for blood to go from a larger to a smaller artery at one angle as another, that such was not the case with a foreign body or clot. Hence he was not disposed to retract what he had said in regard to a clot passing into the left carotid on account of the angle at which it was given off from the aorta, in preference to the right.

Dr. Miller: A floating body follows the current.

Academy adjourned.

---

Atlanta, Jan. 16, 1877.

Vice-President, Dr. J. M. Boring, in the chair.

Dr. Calhoun was called to Marietta to see, in consultation, a patient eighteen years of age, who had suffered for ten days with a cold, partial deafness, and sore throat. During the attack he got wet, which caused an exageration of all the symptoms. The family physician had given the ordinary remedies. The second or third night before he was seen by Dr. Calhoun, he had high fever, accompanied by delirium. The symptoms were those of brain or meningeal trouble. Next day better, but that night again worse, followed on the succeeding day by paralysis of left side of the face, including the lower lid. The muscles of the ball were not affected. Saw him after dark; he was feverish and was excessively drowsy; complained of earache, especially in front of tragus, and was deaf. With an otoscope found in the external meatus a bulging substance, resembling the distended drum. Decided to open by free incision, which was followed by pus and a peculiar substance resembling cerebro-spinal fluid. Another incision caused the escape of similar substance. About one drachm of pus and fluid escaped. He thinks the thin lamina of bone which separate the middle ear from brain, or interior of cranium, was by pressure and inflammation absorbed, and allowed the escape of cerebro-spinal fluid which followed the pus. Gave tonics and advised cleanliness. In four or five days the hearing was tolerable; in five weeks normal; but the paralysis of muscles supplied by portio dura continues. The prognosis as to the paralysis is uncertain. Strychnia and electricity are being given. Has no doubt but that many cases of so-called brain fever in children are nothing more than inflammation of middle ear, especially when attended by ear-ache.

Dr. Baird: Did the orbicularis oculi escape paralysis?

Dr. Calhoun: Yes; all but pal-prebal portion.

Dr. Baird: Thought the symptoms referable to the brain might be otherwise accounted for than by supposing that organ or its meninges inflamed. The fever may have caused the delirium, as is often the case. The paralysis was certainly not dependent on brain lesion. The fluid was more likely to have been cerum, effused in the swollen tissues around the abscess.

Dr. Calhoun: But the escape of fluid continued four or five days after the incision.

Dr. W. F. Westmoreland asked how much fluid escaped in all.

Dr. Calhoun: In all, but not at once, fully half a drachm. The tissues were not swollen after three or four days. The fever, he thinks, was not sufficient to cause the delirium. Is satisfied that the meninges bordering the bone were involved by contiguity of tissue, etc. The delirium was intermittent in character.

Dr. Baird said the delirium of meningitis was not intermittent.

Dr. W. F. Westmoreland does not think the patient ever had circumscribed meningitis, or the subsidence of the symptoms would not have been so rapid as in Dr. C.'s case. The slight fever sometimes necessary to cause delirium precludes the idea of a high grade of fever being present necessarily. Has himself become delirious from an ear-ache lasting for only twelve hours. The fluid, he thinks, was an effusion in the submucous cellular tissue. In uvulitis he has often noticed such a discharge. He regards the continuation of flow for several days as confirmatory of his view.

Dr. Calhoun thought the irritation set up in the meninges was circumscribed, and only spreading as a larger surface of bone became involved. Before a fresh invasion or larger zone of membrane was implicated the system would become accustomed to it, thus causing intermission in the symptoms.

Dr. Baird: An inflammation of the meninges would not spread in successive zones, but would gradually deepen.

Dr. W. F. Westmoreland reported a case, at once rare, unique, and terrible. Ten days ago was called to see a man in Cobb county, who had received an oblique wound in the abdomen, commencing at the median line and cutting the rectus abdominis muscle in twain. The extent of incision was three and a half inches. The wound had been closed with an ordinary needle and cotton thread; the stitches only going through the skin. It was four days after said operation when he first saw the unfortunate. Two of the stitches had given way. The intestines were only pushed back behind the integument, leaving the muscles between them and the peritoneal cavity. They were inflamed, red, swollen, and exhaled an offensive odor; and there was sero-pus exuding; pulse 110 to 115 per minute. He was almost totally unprepared for the operation, having no anæsthetic or the proper instruments; only a small quantity of silver wire. But it was a case that demanded immediate action, and he acted. As he proceeded in the operation of returning the intestines, he found them partially adherent to the skin and each other, and was one hour in tearing up adhesions and returning intestines. This being done, the quill suture was applied, patient put on opium, and left. The shock was terrible. The man is now out of danger; has had several operations on his bowels; has a good appetite, etc.

Dr. Calhoun asked Dr. Westmoreland if he thought it would be good surgery to enlarge the opening in abdomen and hunt for a wounded intestine.

Dr. Westmoreland: If you knew there was a wound of the intestine—which would be indicated by the escape of feces or a fecal smell—it would not only be advisable, but imperative; for nine cases in ten would assuredly die. He, however, recalled to mind one case where a soldier was wounded in the bladder and rectum, the ball lodging in pelvic bones, in which there was recovery. But this was exceptional.

Dr. Calhoun read the following letter, in which a physician from Alabama describes a very singular case:

"I must give you an account of a case of oblique inguinal strangulated hernia, which on the 9th of last month I was called to see. The strangulation had existed eight days at the time I was called to the case. I say nothing about the symptoms from the commencement of his trouble but the condition he was in when first seen by me, only that he was a young man, twenty-three years old, stout and healthy up to his present sickness; has a wife and one child.

Condition he was in when seen—whole abdomen distended very much; in tympanitic condition, and tender to pressure. Had not had an action from the bowels since taken. Tongue red all over; rather of a livid cast, though not entirely dry. Thirst great from the commencement; also efforts to vomit, and extreme sickness of stomach; and for the last two days has been belching and throwing up fecal or stercoraceous matter, the scent of which is very offensive. Has very little fever at present, and he says the great pain which he had in the lower part of the belly on the left side has pretty much ceased. The scrotum on the left side was swollen very much, so much so that you could barely feel the testicle behind the contents of the tumor; and skin of scrotum of a dark livid color, and very tender to handle; the neck of tumor running up into the external abdominal ring, and bulging the muscles a little above the external ring, and is very tender around the neck of tumor.

This is the condition in which I found him. He was very anxious to know if I thought I could relieve him. I could promise him nothing flattering in his then condition. I placed him on an inclined plain, heels up, thighs flexed on belly, head raised and pillows placed at head and support to his shoulders. Applied warm flannels to scrotum, and manipulated the tumor by compression in the hands some twenty-five or thirty minutes, with but little effect. The scrotum was reduced a little, but no effect was produced on the neck at the ring. Just before he was taken from the inclined plain he threw up a quart or more of fecal or stercoraceous matter, which reduced the tympanitic condition of the belly to some extent. He was placed in bed and given an anodyne, and rested pretty well during the night. Next morning an opening occurred in the upper portion of scrotum, and an ounce or two of fecal matter passed out at the opening. I advised poultices to parts, and left. Returned in four days. All left side of scrotum, skin, testicle and all, had sloughed off, and two or three inches of bowel were protruding from the ring in a putrefying condition.

History as given me on this visit on Monday after I left on Saturday. Monday had an action by rectum, and they had thought and hoped, from the upper bowel; but on Tuesday night had a large operation through the artificial opening, and it was passing more or less that way since.

Condition second visit.—Had rested pretty well since I saw him last. The symptoms of peritonitis gradually giving way; appetite improving; sickness of stomach had left him, and he was in no pain; only occasional pains would run up the abdomen from the neck of the stricture. The abdomen somewhat tympanitic, but nothing like it was when first seen. Pulse on this visit numbered 58 only per minute, though full, and he seemed rather indifferent. Poultices to be kept up; diet of a fluid nature, though nourishing.

I left, supposing sooner or later he would die; or if he escaped death, would have an artificial anus through the opening; and they were to send me word how he was doing.

Two weeks later I visited him again—four days since, and found him doing well; all tympanitis of abdomen disappeared, and in a rather collapsed condition. Appetite keen, and last eight or ten days having operations daily by the rectum, and those by the artificial opening ceased entirely. Scrotum nearly healed, and the bowel that protruded from the neck of the stricture on second visit came off in a few days after I left, and at present nothing but pure pus is coming from the opening, and but very little inflammation about the neck of where the tumor was in the lower portion of the abdomen. Says he feels like he could be up going about; and he undoubtedly will get well, from present indications.

I have been practicing medicine thirty years, and this is the first case of strangulated inguinal hernia that has ever come under my observation."

Dr. Westmoreland said he should have acted very much as did the gentleman, unless he could have found both ends of intestine; in which case he would have cut off the gangrenous portion, and stitched the two ends together.

Dr. Baird asked if stricture of intestine was not likely to result after the two ends had been joined, etc.

Dr. Calhoun said Hyrtl, a German surgeon, had once removed a foot of gangrenous intestine, the patient recovering, and years after died of cholera. A post-mortem showed no stricture, but perfect adhesion.

Dr. McIntosh said that an indurated ring was the only mark left in the intestine of a hog which was killed months after losing a foot or more of intestine. Flax thread was used to connect the two ends.

Dr. Westmoreland: Experiments on dogs did not give stricture as a sequelæ; the dogs being killed four months after. This experiment was had with a view to determine which was the better, silver wire or silk, to sew wounded intestines.

Academy adjourned.

---

ATLANTA, Jan. 23, 1877.

Vice-President, Dr. John M. Boring, in the chair.

Dr. J. G. Westmoreland called attention, in a general way, to the prevailing disease at present with us—tonsilitis. Asked what was the usual and proper treatment to prevent the formation of abscess. His observation favored local depletion; thought it better than any topical application, such as iodine, nitrate silver, chlorate potash, etc. Counter-irritation he has found to be of no avail. Scarifies the tonsil in several places, and encourages bleeding; following with no local application. Sometimes, when fever is high, indicating violent inflammation, he gives an emetic as a relaxing agent.

Dr. Baird inquired of Dr. W. why he gave the emetic.

Dr. Westmoreland answered, for its relaxing effect. It is a well known fact that nauseants have great influence in controlling inflammations, wherever situated.

Dr. Griggs asked if he ever gave quinine.

Dr. Westmoreland: No.

Dr. Baird said while he approved of the scarification, and thought he had seen good effects from it; considered it unnecessary. Chlorate of potash relieves the disagreeable dryness, is soothing, and in mild cases, with quinine, meets all the indications. He thought a man with tonsilitis generally needed something to stiffen him up, rather than relaxation.

Dr. Westmoreland: Why do you give quinine?

Dr. Baird: Can't explain how it acts in tonsilitis any more than in other affections. It may counter-act a certain morbific epidemic influence, which certainly exists in the atmosphere during the prevalence of all epidemic or endemic affections.

Dr. Westmoreland: The fever is caused by the tonsilitis. If quinine should stop the fever, what good would have been accomplished, the inflammation continuing? Thought Dr. Baird commenced at the wrong end. Remove the cause and the effects will disappear without interference.

Dr. Baird: If tonsilitis causes fever, what causes tonsilitis?

Dr. Westmoreland: Generally, vicissitudes of the weather, dampness of the atmosphere, with cold, etc.

Dr. Baird: Even should your case go on to suppuration, would you not then give quinine?

Dr. Westmoreland: No; can't see how quinine could expedite the escape of pus or cause the line of demarkation during sloughing to be any more distinct or perfect than nature would have it.

Dr. Baird: Do you not give supporting treatment?

Dr. Westmoreland: Yes; I give the patient plenty of good rich victuals, after the inflammation subsides.

Dr. Griggs asked Dr. W. if he thought the patient would eat rich food with a fever; would they have an appetite for such aliment?

Dr. Westmoreland: I did not start out on putrid sore throat, but even should it occur would not give quinine.

Dr. Boring said it had been his habit to give quinine and tinct. chloride of iron. It was considered by many as a specific.

Dr. Baird said it was very convenient to speak of cold and dampness as a cause of disease, but there was undoubtedly some more tangible cause than them; for how often is it the case that the weather is inclement and cold, and no epidemic prevails. Suggested bacteria as a probable cause.

Dr. W. F. Westmoreland, at the mention of bacteria started to his feet, and dilated beyond the reporter's ken, on infinitesimal germs, thermometrical, barometrical, hygrometrical, and anemometrical influences. Frequent mention of morbific epidemic influences were also made.

Dr. J. G. Westmoreland, unawed and unconvinced, calmly remarked that bacteria and all relating to it were bosh and humbug. If your war is on bacteria, scatter quinine in the air.

Dr. Calhoun: Chronic inflammation of the tonsils leads to hypertrophy, and its effects upon the hearing and speech had been of late a subject of special study with him; especially its effects upon the hearing, which was influenced in various ways. The tonsil, as it enlarges, grows in all directions, especially upwards and backwards, thus closing the eustachian tube; and that tube also takes on inflammation itself, which extends to middle ear, constituting non-suppurative inflammation of middle ear, ordinarily called catarrh of ear. The speech is affected by the inflammation; for in hypertrophy of tonsils there is always sub-acute inflammation, extending to the vocal cords; for after removal of tonsil we always find inflammation of the cords, and the ventricles between the true and false cords are filled by mucous membrane puffed up with inflammatory deposit. Hypertrophied tonsils also affect the teeth, by compelling the sufferer to sleep with the mouth open. This causes the enamel of the teeth to crack, and decay ensues. The treatment *par excellance* is excision of that portion of tonsil protruding beyond the arches of palate.

Hypertrophy is sometimes congenital. Knows of such a case, where the child neither spoke nor heard until it was four or five years old. At ten years of age had one tonsil excised, and there was thirty or forty per cent. increase in both functions. He uses bull-dog forceps and blunt-pointed bistoury. Objects to tonsilitome for various reasons, among which were the difficulty and time taken in adjusting them to tonsil. The hemorrhage has never been excessive in any case where he had operated. In one case it was pretty free, but was easily controlled by common salt.

Dr. W. F. Westmoreland said all hypertrophied tonsils were not the result of inflammation; often congenital, or caused by over-assimulation. He uses Chesignac's tonsilitome always. The old-fashioned instruments for excision are bad, and to be condemned. Observed that the tonsils are pear-shaped bodies, with smallest part next to point of attachment. Two-thirds is a sufficient amount to remove. To avoid hemorrhage, be sure and cut far enough back to escape the point of greatest circumference of these bodies, as the amount of bleeding is directly dependent on the area of cut surface. Go behind the bulk, and troublesome bleeding will rarely occur. In reply to question as to the danger of wounding the carotid, he replied that adhesive inflammation sometimes glued the tonsil and artery together. But even then, with tonsilitome, or operating without it, if you cut from below, having the edge of knife turned toward the uvula, there was no danger of wounding the carotid.

Dr. W. F. Westmoreland presented a pathological specimen in the shape of a multilocular ovarian tumor, extracted post mortem. The woman was twenty-two years old. A tumor was first discovered three years ago. Fifteen months since came under his treatment. She never could be gotten in a condition where he deemed it safe to operate. Dr. J. M. Johnson had for several months been giving her muriate ammonia internally, and applying it externally. She has been tapped regularly every two weeks for the past eight months. Eight gallons of semi-solid, gelatinous, amber colored liquid were evacuated at first tapping; about four gallons each time thereafter. He estimated that the tumor and contents would weigh forty pounds. The last tapping was two weeks prior to death. Post-mortem examination of cyst showed that death was the result of inflammation of the cyst walls themselves; there was no peritonitis. Twenty-four hours after the operation of tapping she had a chill, followed by nausea, vomiting and fever, which continued to dissolution. The congestion in cyst, which went on to inflammation, was caused by relaxation of cyst walls, consequent upon the evacuation of the fluids. He called particular attention to the thickness and color of contents of those cysts which had not been evacuated by tapping. Said about forty pounds of it per month had been drawn off for the past eight months. The tapping had to be done above the umbilicus in each instance; the trochar inserted at the usual place below, caused but little fluid to escape.

Academy adjourned.

